# Trends and developments in oral health literacy: a scientometric research study (1991–2020)

**DOI:** 10.1038/s41405-021-00066-5

**Published:** 2021-03-01

**Authors:** Yue Sun, Chunying Li, Yan Zhao, Jing Sun

**Affiliations:** 1 Department of Community Nursing, School of Nursing, Peking University, Beijing, China; 2 Peking University Health Science Library, Beijing, China; 3 School of Biomedicine, Beijing City University, Beijing, China

**Keywords:** History of dentistry, Preventive dentistry, Dental education

## Abstract

**Objective:**

This study aimed to establish the current situation, intellectual base, hotspots, development trends, and frontiers of oral health literacy (OHL) from the literature.

**Methods:**

We analyzed 1505 bibliographic records dated between January 1990 and December 2020 retrieved from the Web of Science Core Collection and the Scopus database. We used CiteSpace for word frequency analysis, co-occurrence analysis, co-citation analysis, clustering analysis, and burst analysis.

**Results:**

The total number of publications increased year-on-year, with the majority of publications coming from the USA. Most studies focused on the relationship between (oral) health literacy and oral health, and the development of OHL instruments. The top 10 keywords by frequency were “health literacy”, “oral health”, “attitude to health”, “dental caries”, “adult”, “children”, “dental care”, “knowledge”, “questionnaire”, and “adolescent”. The keyword with the highest burst intensity was “dental health education”.

**Conclusions:**

OHL research is a thriving field. The field is focused on the development of an OHL instrument and health promotion practice. Strategic cooperation among countries, institutions, authors, hospitals, and communities will be important to encourage further OHL research and address oral health problems.

## Introduction

The World Health Organization (WHO) (2016) suggested that oral health is a key indicator of overall health, well-being, and quality of life.^1^ It can influence people’s lives, and cause pain, discomfort, disfigurement, and even death.^2^ Oral diseases are highly preventable, but remain common in many countries.^3^ The Global Burden of Disease Study 2017 estimated that oral diseases affect 3.5 billion people worldwide, with untreated dental caries being among the most prevalent noncommunicable diseases.^3^

Oral health literacy (OHL) is the “degree to which individuals have the capacity to obtain, process and understand basic oral health information and services needed to make appropriate health decisions”.^4^ It is therefore about individual capacity to understand and use dental information to transform oral health behaviors. Low OHL limits the capacity to understand dentists’ instructions, which hinders the maintenance of oral health.^5^

Many studies have shown that there is strong evidence linking oral health status with OHL.^6,7^ Baskaradoss^6^ found that more than a third of people with limited OHL had high periodontal risk, compared with about 7% of those with adequate OHL. Das et al.^8^ found that 60% of research participants with periodontal pocket formation had low OHL, compared with just 36% among those with good OHL. In another study, people with limited OHL had poorer periodontal health.^5^ Low parental OHL was associated with dental caries among children.^9^ Improving OHL may help to increase adherence to medical instructions, and improve self-management skills and overall treatment outcomes.^6^ OHL should therefore be a priority in oral health promotion programs as a determinant of oral health.^10^

Since OHL was first defined by the US Department of Health and Human Services, there have been numerous publications and studies in the field, and our understanding of OHL has advanced significantly. However, there has been no review that reveals the diversified content of OHL. Therefore, it is particularly important to use bibliometric methods to quantitatively analyze the characteristics of the literature in the field of OHL and to objectively evaluate the subject’s research status and development history.

Scientometrics refers to the study of measuring and analyzing scientific literature.^11^ Scientometrics is the analysis of the general characteristics of research and development (R&D) output, research hotspots, intellectual structures, and the R&D capability of countries in a certain field, using information from papers and patents such as titles, keywords, abstracts, and texts. The methods include the analysis of word frequency, citations, authorship, co-citations, co-authorship, co-words, and counts of authors, research groups, and countries.^12^ CiteSpace is one of the most popular analysis tools, and plays an important role in scientometrics to explore the core structure, development history, hotspots, and overall knowledge structure of a discipline.^13,14^

To date, no scientometrics analysis of OHL has been performed. Our study aims to generate visualized knowledge maps of OHL and analyze the current situation, intellectual base, hotspots, and development trends. We hope to provide an insight into OHL and a basis for future research.

## Method

### Data sources

Literature was retrieved online through the Web of Science Core Collection (WoSCC) and the Scopus database. We used the search string in WoSCC: TS = (“oral health literacy” OR “oral health literate” OR “oral medical literacy” OR “oral medical literate” OR “oral health knowledge” OR “literacy in dentistry” OR “Estimate of Literacy in Dentistry” OR “Test of Functional Health Literacy in Dentistry” OR “Oral Health Literacy Instrument” OR “Oral Health Literacy Assessment” OR “Comprehensive Measure of Oral Health Knowledge” OR “Oral Health Literacy Adults Questionnaire”) OR (TS = (“patient medical knowledge” OR “patient understand knowledge” OR “health literacy”) AND TS = (oral OR dentistry OR dental* OR Periodont*)); and in Scopus: (TITLE-ABS-KEY (“oral health litera*” OR “oral medical litera*” OR “oral health knowledge” OR “literacy in dentistry” OR “Estimate of Literacy in Dentistry” OR “Test of Functional Health Literacy in Dentistry” OR “Oral Health Literacy Instrument”) OR TITLE-ABS-KEY (“Oral Health Literacy Assessment” OR “Comprehensive Measure of Oral Health Knowledge” OR “Oral Health Literacy Adults Questionnaire”)) OR (TITLE-ABS-KEY (“patient medical knowledge” OR “patient understand knowledge” OR “health literacy”) AND TITLE-ABS-KEY (oral OR dentistry OR dental* OR periodont*)). Document type = article; time span: January 1990 to July 2020. There were no language restrictions. The initial sample included 941 articles from WoSCC and 1314 articles from Scopus.

Data from both databases were imported into CiteSpace. Importing the Scopus data required converting ris-files to a text-file format using the software’s import features, whereas the WoSCC data were imported directly into CiteSpace from text files. Duplicate papers were eliminated, resulting in a final sample of 1505 articles.

### Analysis tool

CiteSpace (7.1.R5, 64 bit) was used for analysis. This software generates node link graphs, citation network maps, and other visual results. In the CiteSpace knowledge maps, nodes reflect different elements, such as authors, countries, and cited references. The size of the nodes indicates the number of publications or frequency (i.e., citation count). A larger node shows more publications or higher frequency. The different colors within the nodes represent different times, the connection lines between the nodes reflect the relationship between them, and the color of the line reflects the date of the first cooperation or co-citation.^15^ Centrality is a turning point in a field and is shown by purple on the node ring in the knowledge map. It represents the significance of nodes in a network. The thickness of the purple betweenness centrality trim shows the strength of the betweenness centrality. A key node is defined as a node with a betweenness centrality ≥ 0.1 in the network.^16^ Citation bursts provide a useful means to trace the development of a research focus. Citation rings in red show the time slices in which citation bursts, or abrupt increases in citations, are detected.^17^ Cluster analysis used the clustering function of CiteSpace. The modularity and mean silhouette scores of clustered co-citation networks are two indicators of the general structural properties of a research front. The modularity value (*Q*) measures the extent to which a network can be divided into independent modules, modularity *Q* > 0.3 is convincing. A silhouette value (*S* value) close to 1 indicates that references within a cluster contain highly consistent or similar content.^18,19^ In this study, the parameters of CiteSpace were: time slicing (1991–2020), years per slice (1), term source (all selection), node type (choose one at a time), selection criteria (top 50), and pruning (pathfinder).

## Results

### Trends in the literature

**Fig. 1:**
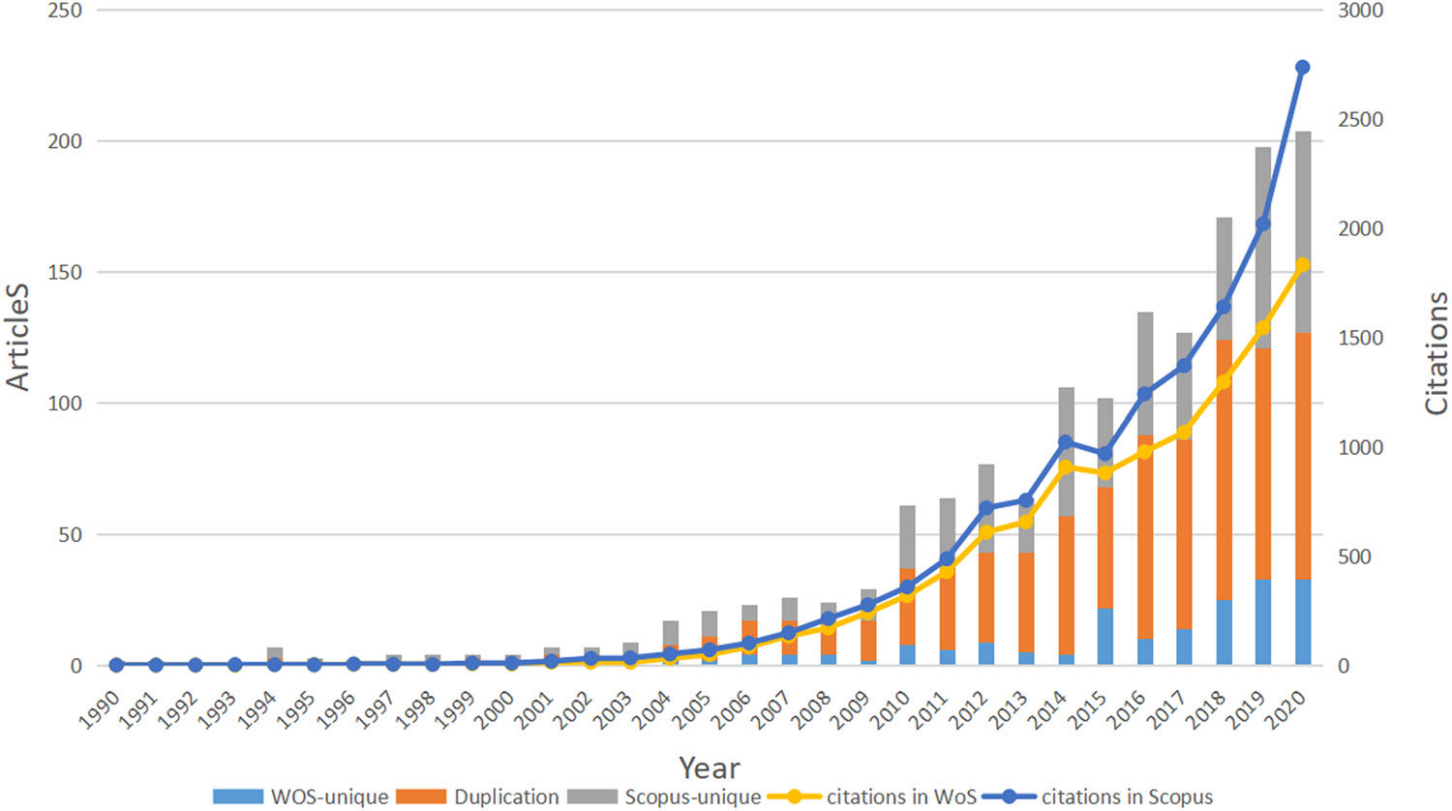
Publication and citation outputs. The annual number of the literature relating to published articles and citations in oral health literacy research from 1990 to 2020.

Figure 1 shows that the literature on OHL relating to published articles and citations grew significantly between 1991 and 2020. For articles, in 2019, 198 published articles were recorded in the databases, six times more than in 2009. From 2016 to 2020, 835 articles were published, 422 more than the 413 published from 2011 to 2015. Citation counts have seen a similar evolution and increase. In 2019, 2019 citations appeared in Scopus and 1543 in WoSCC, compared with 276 and 240 citations in the respective databases in 2009. From 2016 to 2020, 9004 citations appeared in Scopus and 6713 in WoSCC, compared with 3952 and 3480 citations in the respective databases from 2011 to 2015. These results show that the amount of research in this field is increasing, seemingly indicating increasing interest in the field over recent years.

### Analysis of countries

**Fig. 2:**
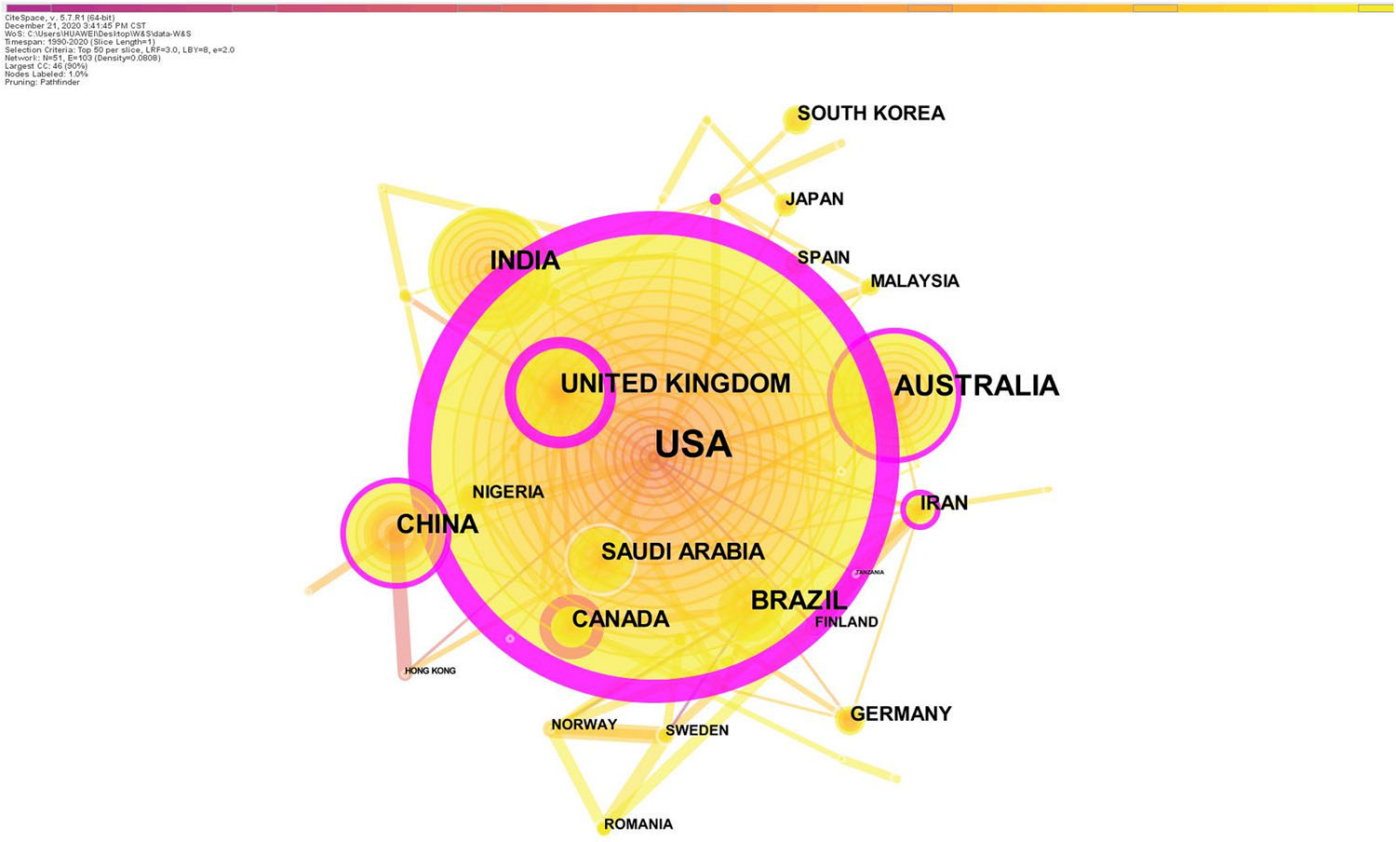
The distribution of countries. The network map of countries related to oral health literacy research.

Generating a country map in CiteSpace gave 52 nodes and 107 links (Fig. 2). Publications on OHL came from 52 countries. The top 5 countries for co-occurrence and centrality are listed in Table 1. The USA contributed the most publications (500), followed by Australia (111) and India (84). The top 3 countries by centrality (≥0.1) were the USA (0.68), UK (0.39), and Canada (0.20). The USA was the most important research source by publication number and centrality. Figure 2 shows extensive collaboration between the USA, Australia, UK, Canada, and Iran. The strongest collaborations were among Brazil, Sweden, and Norway.

**Table 1:** Top 10 countries according to the number of publications and centrality.

Ranking	Country	Publications	Ranking	Country	Centrality
1	USA	500	1	USA	0.68
2	Australia	111	2	UK	0.39
3	India	84	3	Canada	0.20
4	China	69	4	Bangladesh	0.18
5	UK	66	5	China	0.15
6	Brazil	60	6	Iran	0.15
7	Saudi Arabia	45	7	Australia	0.14
8	Canada	36	8	Saudi Arabia	0.09
9	Iran	31	9	Finland	0.09
10	South Korea	24	10	Brazil	0.07

### Analysis of authors and co-cited authors

**Fig. 3:**
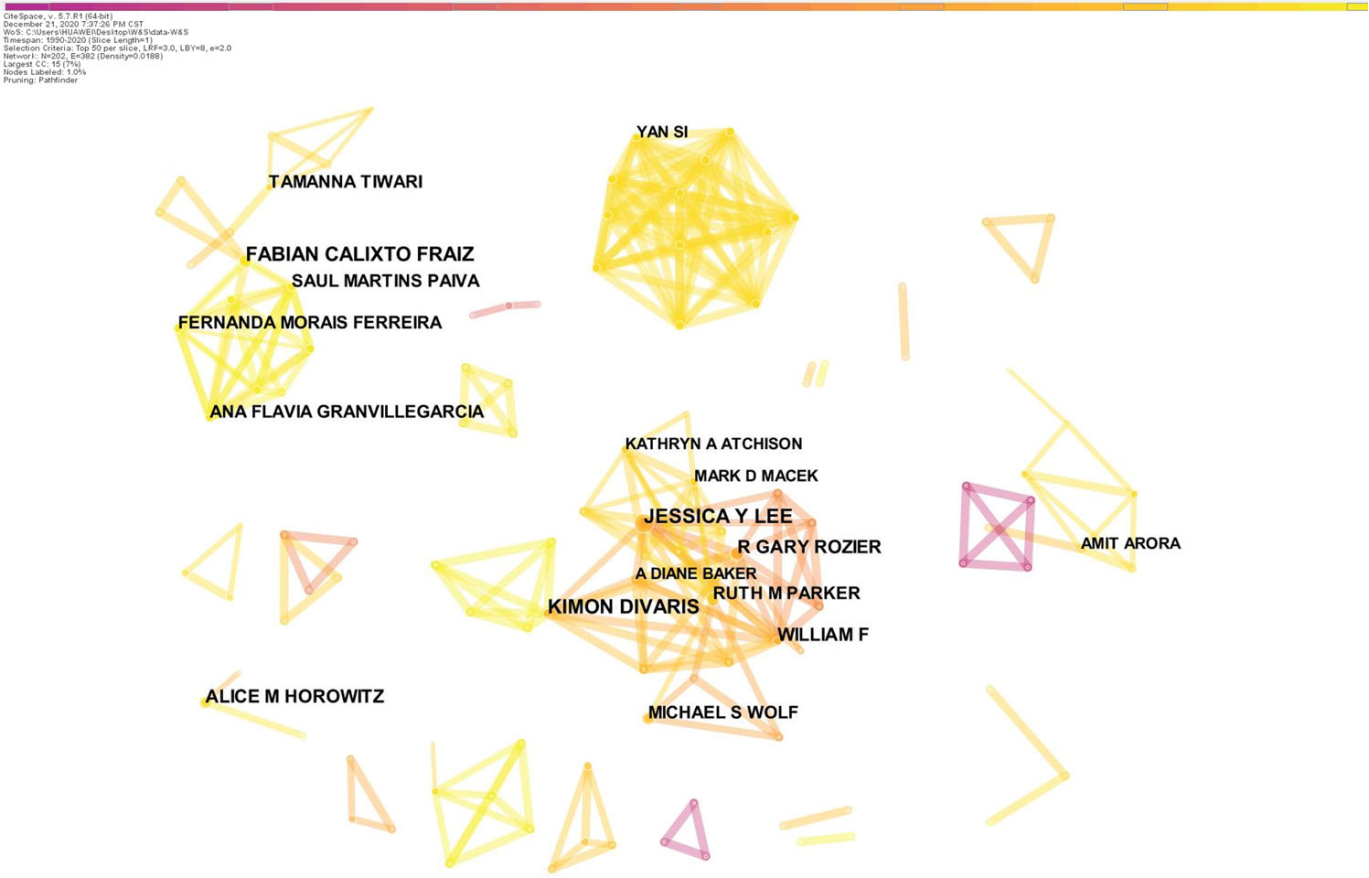
The distribution of authors. The network map of active authors offered to oral health literacy research.

The co-author map showed 202 nodes and 382 links (Fig. 3). The 1505 articles were by 202 authors. The top 5 authors are shown in Table 2. Three large cooperation networks had been formed. There were some collaborations among Jessica Y. Lee, A. Diane Baker, William F. Vann Jr, Kimon Divaris, and R. Gary Rozier, among Fabian Calixto Fraiz, Saul Martins Paiva, Fernanda Morais Ferreira, and Ana Flavia Granvillegarcia, and among Yan Si, ChunXiao Wang, WenSheng Rong, Xing Wang, and BaoJun Tai. No other large collaboration networks had been formed.

**Table 2:** Top 10 authors according to the number of publications.

Ranking	Count	Author	Year	Country
1	11	Jessica Y. Lee	2007	USA
2	10	Fabian Calixto Fraiz	2016	Brazil
3	10	Kimon Divaris	2011	Brazil
4	9	Alice M. Horowitz	2012	USA
5	9	R. Gary Rozier	2007	USA
6	9	William F. Vann Jr	2007	USA
7	8	Fernanda Morais Ferreira	2018	Brazil
8	8	Michael S. Wolf	2010	USA
9	8	Ruth M. Parker	2010	USA
10	8	Saul Martins Paiva	2018	Brazil

**Fig. 4:**
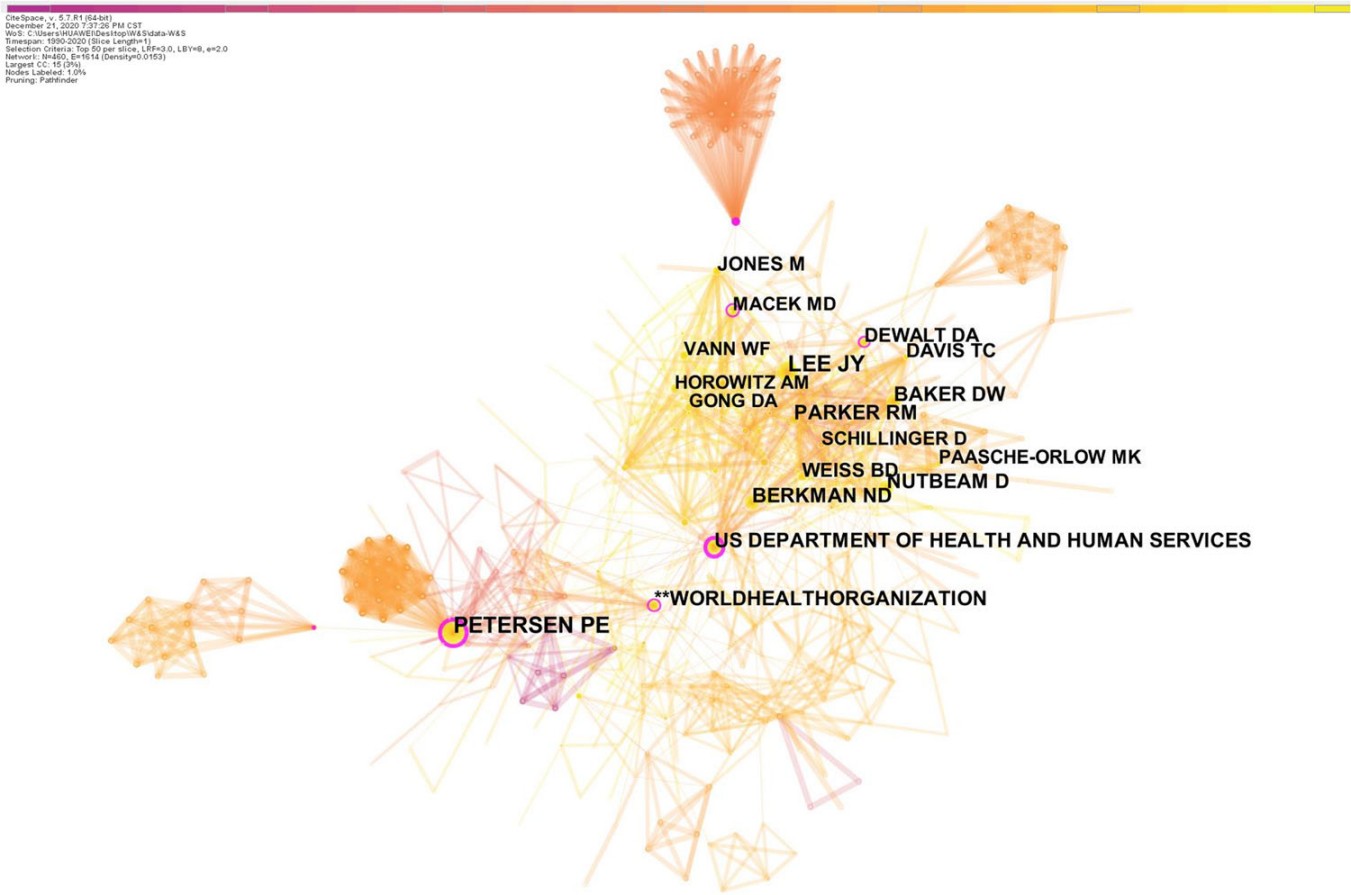
The distribution of cited authors. The network map of co-cited authors offered to oral health literacy research.

The author co-citation map had 460 nodes and 1614 links (Fig. 4). The author with the highest co-citation count was Poul Erik Petersen (161 citations), followed by Jessica Y. Lee (157 citations) and David W Baker (110 citations). Table 3 shows that the top 8 co-cited authors with centrality > 0.1 (“core strength” researchers) were Poul Erik Petersen, US Department of Health and Human Services, Miranda R. Andrus, Darren A. DeWalt, WHO, Mark D. Macek, Ruth Freeman, and Micheala Jones.

**Table 3:** Co-cited authors according to a centrality > 0.1.

Ranking	Centrality	Count	Year	Author
1	0.36	161	2001	Poul Erik Petersen
2	0.35	102	2005	US Department of Health and Human Services
3	0.24	7	2007	Miranda R. Andrus
4	0.17	73	2007	Darren A. DeWalt
5	0.14	95	2006	World Health Organization
6	0.12	70	2011	Mark D. Macek
7	0.11	7	2009	Ruth Freeman
8	0.1	79	2010	Micheala Jones

### Analysis of journals and co-cited journals

**Fig. 5:**
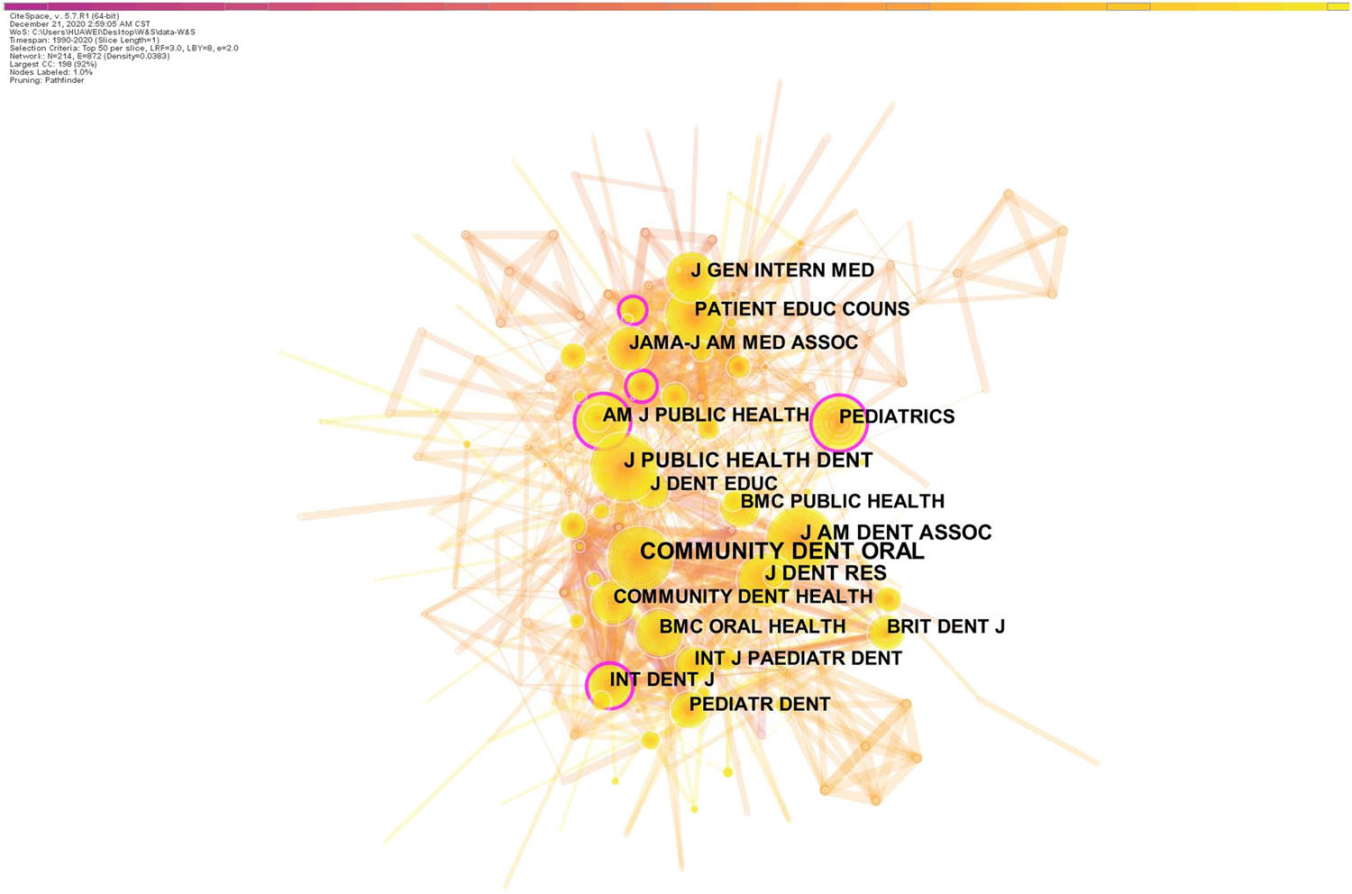
The distribution of cited journals. The network map of co-cited journals related to oral health literacy research.

The co-citation journal map had 214 nodes and 872 links (Fig. 5). More highly cited journals included *Community Dentistry and Oral Epidemiology* (2019 impact factor (IF): 2.135), the *Journal of Public Health Dentistry* (2019 IF: 1.743), the *Journal of the American Dental Association* (2019 IF: 2.803), the *Journal of Dental Research* (2019 IF: 4.914), and *BMC Oral Health* (2019 IF: 1.911). Table 4 shows the top 5 co-cited journals by centrality (≥0.1). The journal with the highest centrality was *Social Science & Medicine* (2019 IF: 3.616), followed by the *American Journal of Public Health* (2019 IF: 6.464).

**Table 4:** Top 5 co-cited journals according to centrality.

Ranking	Centrality	Count	Year	Journal	Impact factor
1	0.20	122	2004	*Social Science & Medicine*	3.616
2	0.16	222	2002	*American Journal of Public Health*	6.464
3	0.15	212	1994	*International Dental Journal*	2.038
4	0.12	210	2004	*Pediatrics*	5.359
5	0.12	104	2005	*Annals of Internal Medicine*	21.317

### Analysis of the intellectual base

**Fig. 6:**
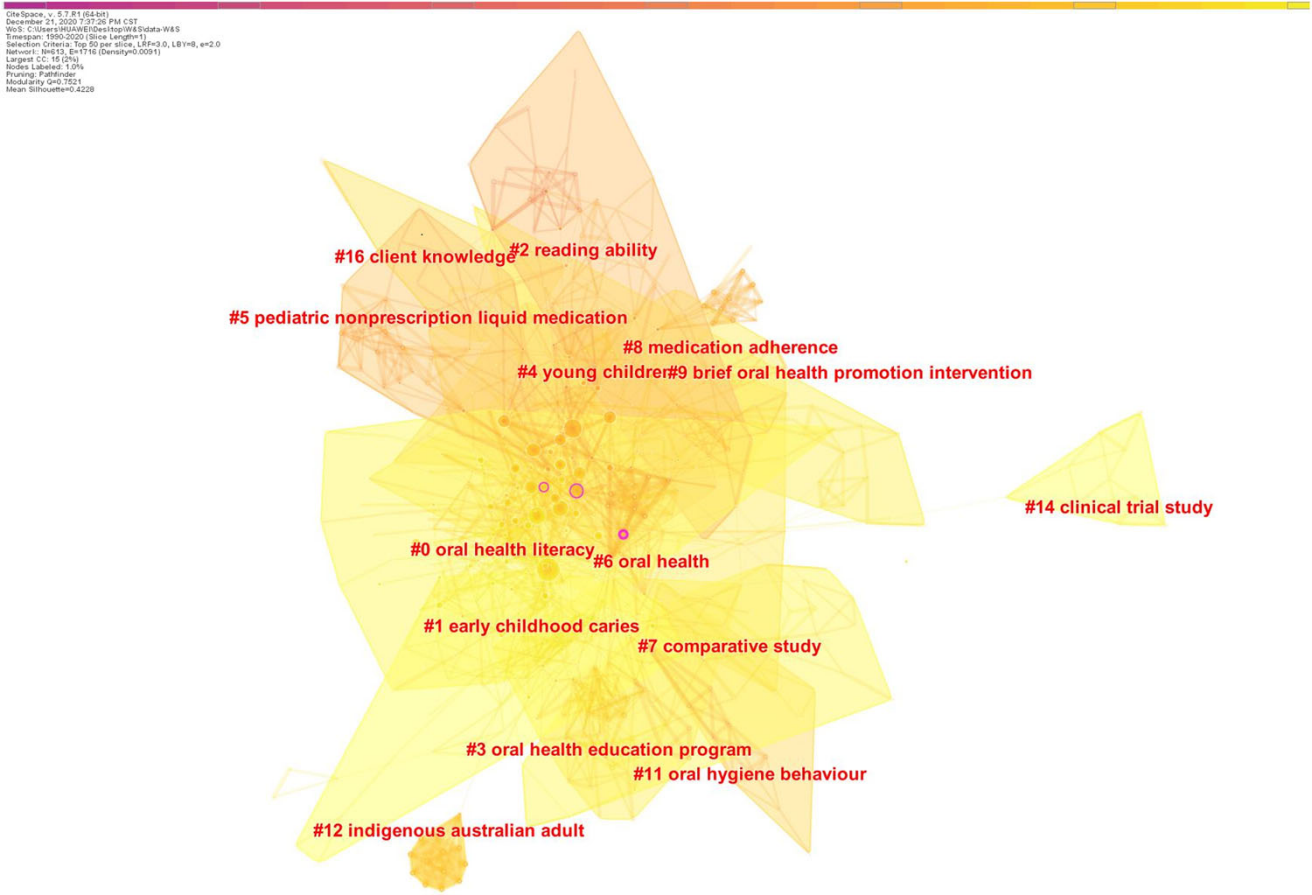
The clusters of cited references. The co-citation cluster map of references from publications in oral health literacy research.

The co-citation reference map had 613 nodes and 1716 links (Fig. 6). Table 5 lists the top 5 co-cited references by centrality. The cited article with the highest centrality was by Jessica Y. Lee et al.^20^ This study indicated that differences in OHL levels between racial groups persisted after adjusting for educational attainment and sociodemographic characteristics.

**Table 5:** Top 5 co-cited references according to centrality.

Ranking	Centrality	Cited references	Author (ref.)	Cluster
1	0.21	Oral health literacy levels among a low-income WIC population	Lee et al.^20^	4
2	0.13	Oral health literacy: a pathway to reducing oral health disparities in Maryland	Horowitz and Kleinman^32^	0
3	0.11	Impact of caregiver literacy on children’s oral health outcomes	Miller et al.^33^	4
4	0.1	Update on health literacy and diabetes	Bailey et al.^34^	13
5	0.1	Health literacy and public health: a systematic review and integration of definitions and models	Sorensen et al.^35^	1

The clustering of similar references resulted in co-citation clusters. The modularity (*Q* value) was 0.7521, and the *S* value was 0.4228. We used the local linear regression (LLR) algorithm to label the clusters. Figure 6 shows the co-citation reference clusters. In total, 14 clusters were identified, including 6 major clusters (Table 6). Cluster #0, which was labeled “oral health literacy”, contained 110 references (*S* value = 0.798) about OHL status and the relationships among OHL, oral health behavior, and oral health status. Cluster #1 was labeled “early childhood caries” and contained 44 references (*S* value = 0.873), and was mainly about the prevention of early childhood caries and parental health literacy influence. Cluster #2 was labeled “reading ability” and contained 41 references (*S* value = 0.863), and was mainly about health literacy or functional health literacy. Cluster #3 was labeled “oral health education program” and contained 39 references (*S* value = 0.909), and was mainly about oral health care programs, especially for pregnant women. Cluster #4 was labeled “young children” and contained 37 references (*S* value = 0.889), and was mainly about the impact of caregiver literacy on children’s oral health outcomes and the development of an OHL instrument. Cluster #5 was labeled “pediatric nonprescription liquid medication” and contained 37 references (*S* value = 0.877), and was mainly about literacy and the misunderstanding of prescription labels. The top 5 co-cited references by the number of citations and centrality were mainly from these six clusters.

**Table 6:** Major reference co-citation clusters.

ID	Size	Mean silhouette	Mean year	Terms (local linear regression)
0	110	0.798	2013	oral health literacy (118.96); oral health behavior (59); oral health knowledge (41.97); pilot study (41.74)
1	44	0.873	2015	early childhood caries (73.41); future intervention preference (62.85); qualitative study (53.8); parental perspective (53.8)
2	41	0.863	2004	reading ability (55.99); patients’ literacy skill (55.99); aural literacy (49.71); intended medication adherence (43.44)
3	39	0.909	2011	oral health education program (48.09); oral health recommendation (39.98); nationwide survey (39.98); dental service (31.9)
4	37	0.889	2007	young children (70.69); caregivers’ assessment (45.68); national survey (40.39)
5	37	0.877	2006	pediatric nonprescription liquid medication (47.47); measuring device (47.47); dosing direction (47.47); parents’ medication administration error (40.94)

### Analysis of hotspots

**Fig. 7:**
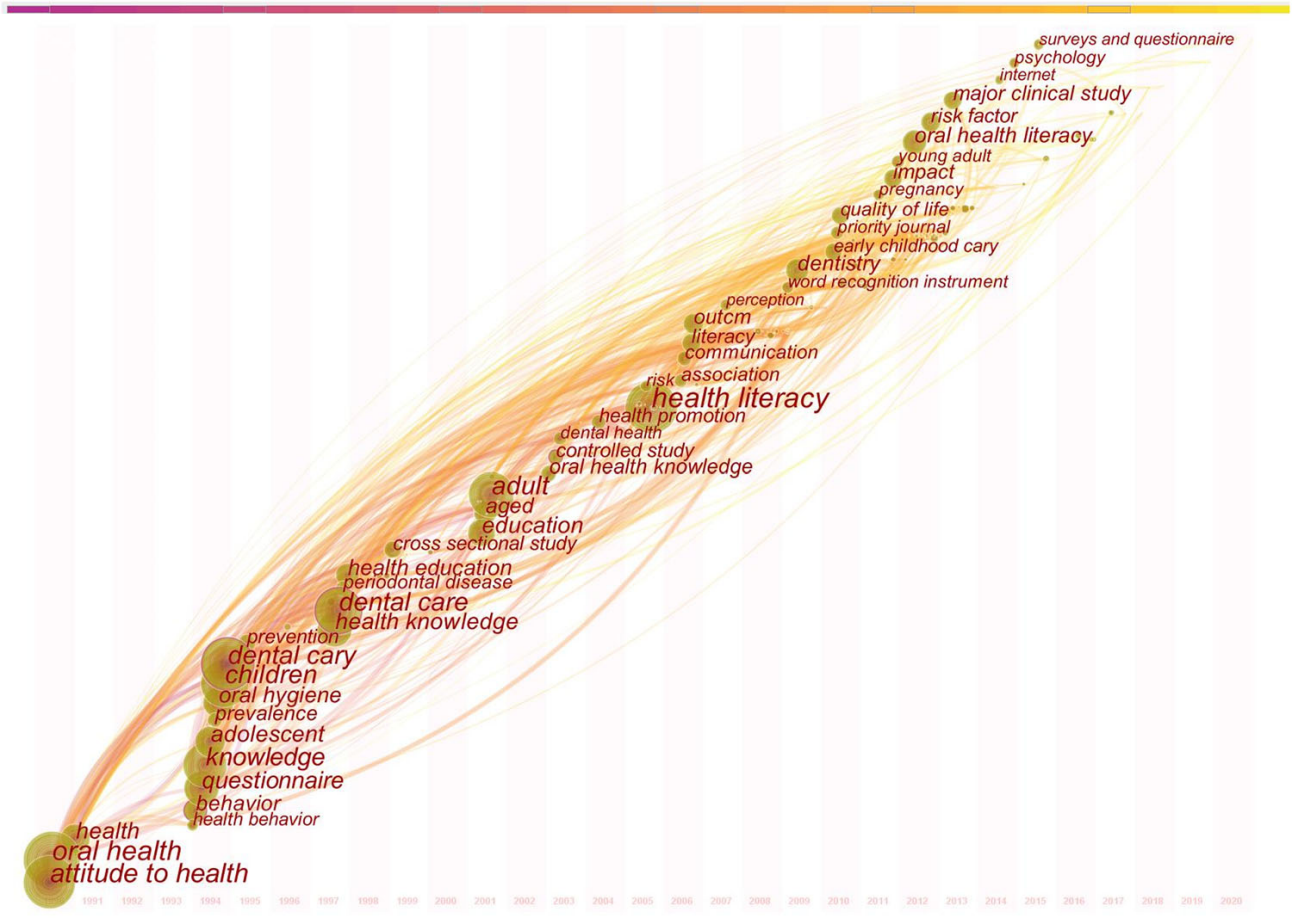
The time zone view of keywords. The time zone view of co-occurring keyword from publications in oral health literacy research.

Figure 7 presents the time span of the most frequently occurring keywords and their respective co-occurrence links. “Oral health”, “health”, and “attitude to health” were three of the earliest keywords. Over time, “knowledge”, “dental caries”, “oral hygiene”, “dental care”, “adult”, “health literacy”, and other keywords developed. More recently, “impact”, “risk factor”, “internet”, “psychology”, and “surveys and questionnaire” emerged. New keywords for 2019 and 2020 included “community”, “human experiment”, and “validity”.

Table 7 shows the top 10 keywords by frequency and centrality. The keyword with the highest frequency was “health literacy”, followed by “oral health”. The keyword with the highest centrality was “controlled study”.

**Table 7:** Top 10 keywords according to frequency and centrality.

Ranking	Keyword	Frequency	Ranking	Keyword	Centrality
1	health literacy	415	1	controlled study	0.13
2	oral health	384	2	behavior	0.12
3	adult	239	3	dental care	0.12
4	knowledge	207	4	adolescent	0.1
5	dental caries	165	5	health literacy	0.09
6	children	139	6	patient education	0.09
7	questionnaire	133	7	health	0.08
8	attitude to health	124	8	child	0.08
9	care	123	9	age	0.08
10	attitude	122	10	adult	0.07

**Fig. 8:**
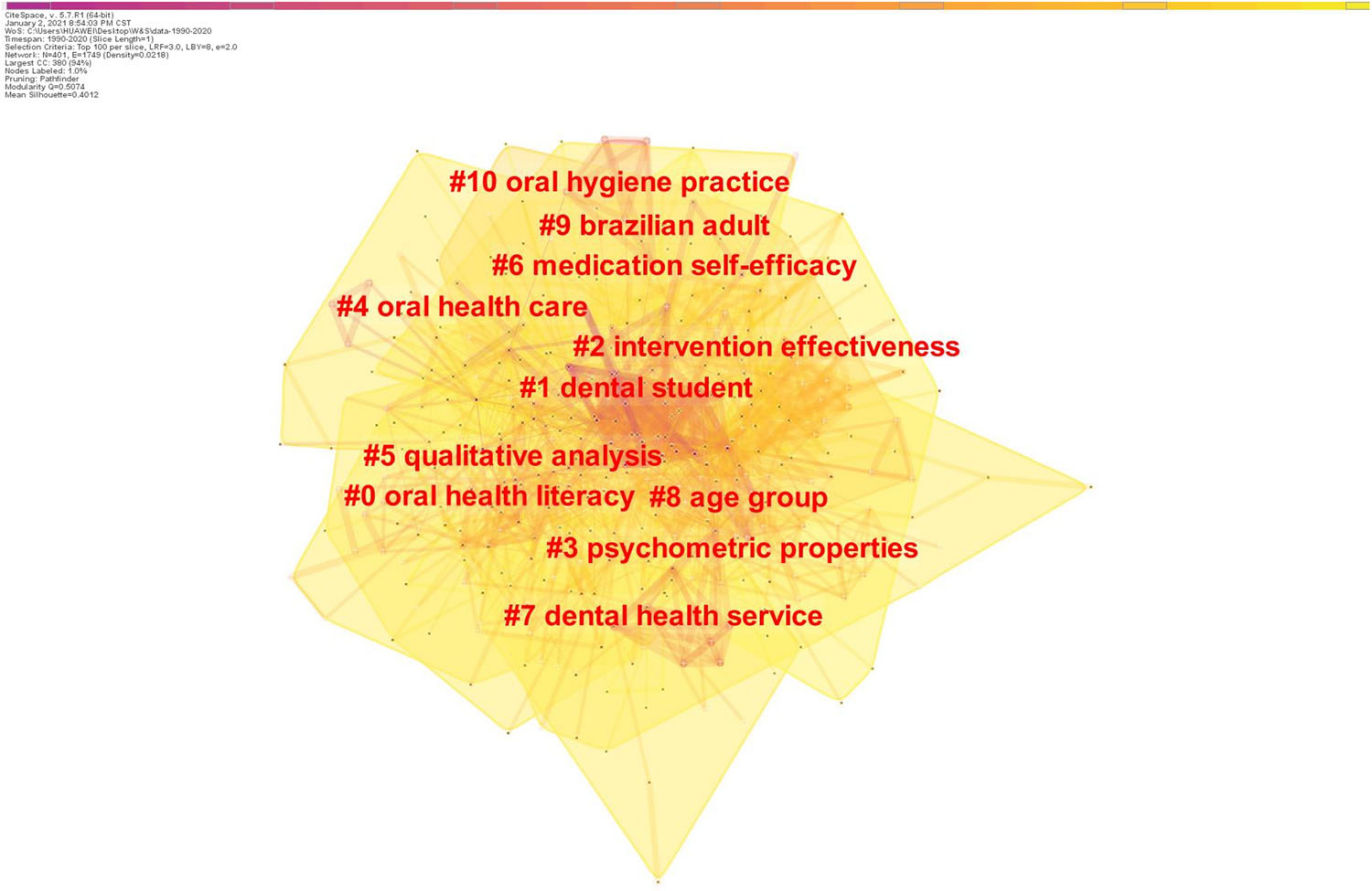
The clusters of keywords. The co-citation cluster map of keywords from publications in oral health literacy research.

Figure 8 shows the co-citation keywords clusters. The modularity (*Q* value) was 0.5074, and the *S* value was 0.4012. We used the LLR algorithm to label the clusters. There were ten keyword clusters, including three major clusters. The largest of the three major clusters, Cluster #0, included 62 keywords and a mean *S* value of 0.685. The literature in this cluster describes OHL and OHL assessment scales. The hotspot focused on the impact of OHL, oral health behavior, or oral health knowledge on oral health status, and the development and psychometric validation of OHL scales. The second largest cluster (Cluster #1) included 59 keywords and a mean *S* value of 0.826. It mainly discussed the oral health knowledge, attitudes and practice status of different populations, and dental education programs, such as information seeking behavior, available health care services, and health communication between dentist and patient. The third largest cluster (Cluster #2) included 56 keywords and a mean *S* value of 0.726. These studies covered factors influencing oral health and the intervention program.

**Fig. 9:**
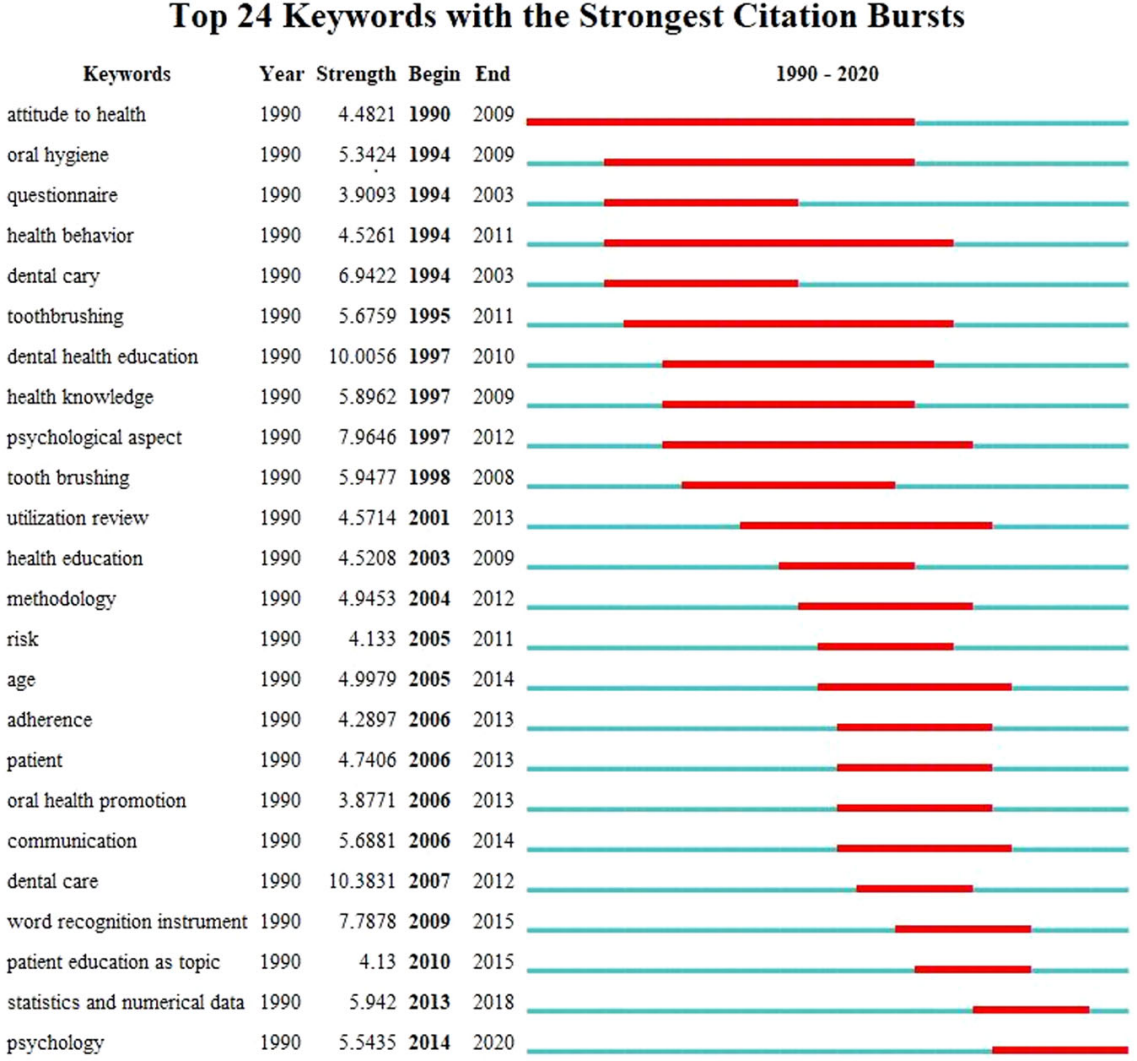
The citation bursts of keywords. The keywords with strong citation bursts from publications in oral health literacy research.

So-called “burst words” are words that are cited frequently over a period of time.^21^ Figure 9 shows the top 24 keywords with their burst impact. The strongest citation burst keywords appeared from 1990 to 2020. “Attitude to health”, “oral hygiene”, “questionnaire”, “health behavior”, and “dental caries” were the earliest burst keywords. “Attitude to health” had the longest duration of burst, from 1990 to 2009. “Statistics and numerical data” and “psychology” were the most recent burst keywords. “Dental care” (strength: 10.3831) had the highest burst intensity.

## Discussion

OHL has become an increasingly important area of oral health research. Our study represents the first attempt to analyze the research on OHL using CiteSpace software. CiteSpace has been widely used to detect and visualize scientific knowledge, trends in the literature, and future directions of research.^16,19^ For example, it was used to analyze publications on the relationship between betel quid chewing and oral cancer from 1998 to 2017.^22^ Other researchers have used it to gain an overview of studies of traumatic dental injuries.^23^ We used CiteSpace to analyze articles on OHL from 1991 to 2020 to evaluate the origin, current trends, and hotspots of research on OHL.

The time distribution and growth trends of academic papers are the most direct manifestation of academic attention and knowledge in this field.^24^ Over the last 30 years, an upward trend has been observed in citations regardless of in WoSCC and Scopus database (Fig. 1). Besides, we identified an increasing number of research publications about OHL, which can be divided into two periods—elementary (1990–2009) and rapid development (2010–2020) (Fig. 1)—indicating that OHL research is likely to continue to increase in the future.

An important output of research is the quantity of publications in peer-reviewed academic journals.^25^ Scientific publication is considered a component of constructing a knowledge-based economy. Better economics and expenditure for scientific research in developed countries may have a positive influence on research productivity and quality.^14^ The USA, an economically powerful country, is a key player in scientific research on OHL, contributing about one-third (500/1505, 33.2%) of total literature. Table 2 shows that a total of 60% of the most productive researchers were from the USA. These findings show that the USA, as a developed country, plays a leading role in the field of OHL research. This is consistent with findings showing that the level of oral health care is closely connected to economic factors.^2^ This study is not the first to show the leading role of the USA in producing and publishing scientific literature.^26,27^

Mapping the countries’ and authors’ cooperation networks revealed an essential class of scientific social networks, showing the key structures of scientific collaboration.^14^ Growing global contacts and exchanges have eroded isolation.^14^ However, our results showed that there was perhaps insufficient cooperation among the countries and authors in this field. For instance, in Fig. 2, 52 nodes and 107 links are shown, meaning that 52 countries produced the literature, with 107 cooperative relationships among them—more links mean more collaboration.^25^ This is consistent with other studies,^22,25,28^ and suggests insufficient cooperation. Figure 3 also showed distinct clusters representing disciplines or colleges. For example, Jessica Y. Lee, Kimon Divaris, R. Gary Rozier, William F. Vann Jr, and A. Diane Baker are all from the University of North Carolina at Chapel Hill. More autonomy, international academic exchanges, and cooperation are therefore necessary to promote the development of the discipline.^28^

The academic influence of authors can be determined by the number of publications and the frequency of citations.^25^ Citation frequency analysis highlighted Jessica Y. Lee, who was involved in research on the development of an OHL instrument and survey, and explored the relationships among OHL, self-efficacy, and oral health status.^20,29–31^ Table 3 also showed several “core strength” researchers, whose studies have had an important influence on the field.^25^ However, few authors had a centrality > 0.1, indicating the lack of an influential core author group in the field of OHL. This result suggested that more research on OHL is needed and more researchers should contribute to this field.^25^

We also identified the core journals publishing on OHL, reflecting the use and influence of articles published in these journals.^25^ The top 5 journals had a centrality > 0.1 (Table 4), indicating that articles published in these journals were of higher quality and greater influence on OHL studies. By combining the citation frequency analysis and centrality analysis, we found that the *American Journal of Public Health*, the *International Dental Journal*, and *Pediatrics* are all highly cited and high-centrality journals. These three journals have published many high quality articles, and their academic influence was higher than other similar journals in the field of OHL.

By analyzing references, we identified the intellectual base in the field of OHL. Studies of key nodes with relatively high centrality are the foundation of the research field.^16^ Articles with high centrality rankings focused on OHL levels, improvement of oral health through OHL, and relationships between health literacy and health outcomes (Table 5).^20,32–35^ The clustering of similar references resulted in co-citation clusters, which could be used to explore the main topics.^19^ By analyzing the co-cited references of the six major clusters (Fig. 6), we found that the intellectual base topics were relationships between health literacy/OHL and oral health, improving oral health care practice programs, and development of an OHL instrument. The 14 clusters were relatively concentrated, non-dispersed, and overlapping, indicating that the topics in the intellectual base are concentrated. The intellectual base in the field of OHL is closely related to the field of health promotion theory and health literacy.

A hotspot is a scientific issue or topic discussed in a group of documents that are linked to a period of time.^25^ An analysis of high frequency keywords can be used to determine research hotspots as well as to monitor the research frontier transitions in a field.^21^ The highest frequency keyword was “health literacy” (Table 7), indicating that OHL was closely related to health literacy. Other high frequency keywords included “oral health”, “adult”, “knowledge”, “dental caries”, “children”, “questionnaire”, and “attitude to health”. We can deduce that there is a focus in the field on the relationship between OHL and oral health in the whole population. Most publications included descriptive and cross-sectional studies, with a lack of randomized controlled intervention studies. These results suggest that there is a need in this field for more intervention research on improving OHL to enhance the oral health level of the entire population.

Detection of a citation burst indicates dramatically increasing literature citation frequencies, which can last for multiple years or a single year.^14^ A stronger burst indicates that there is more focus on this research topic.^14^ The emergence date of burst words shows when topics became the focus of attention. A word with high burst strength may indicate a significant turning point in the field of research.^25^ Analysis of the time zone view (Fig. 7) and the strongest citation burst keywords (Fig. 9) therefore reveals the hot topics and frontiers in different periods.^17^ Before 2010, the total number of keywords was relatively small. Keywords such as “oral health”, “dental caries”, “attitude to health”, “knowledge”, and “health education” appeared first. The strongest citation burst keyword was “dental health education”, a topic of early attention in this field.^36,37^ In 2000, OHL was first defined in the US Department of Health and Human Services policy, Healthy People 2010,^38^ and since then there have been more studies about the association of health literacy with oral health status.^39–41^ Subsequently, keywords such as “health promotion”, “health literacy”, and “association” appear successively. There was rapid growth from 2010 to 2020 in several areas, including dental care and OHL instruments. “Dental care” and “word recognition instrument” had the highest burst strength during this time, demonstrating the focus of attention in this field. In 2007, researchers developed a word recognition instrument to test health literacy in dentistry: the REALD-30.^29^ Reports released by the American Dental Association (ADA) and the US Institute of Medicine’s (IOM) also underpinned the importance of OHL. In 2009, the Health Literacy in Dentistry Action Plan 2010–2015 was released by the ADA,^42^ and in 2011, the IOM released a report on advancing oral health in America.^43^ Bursts also showed that oral health education and OHL instruments continued to develop. Keywords such as “early childhood caries”, “pregnancy”, “young adult”, “impact”, “risk factor”, “psychology”, “surveys and questionnaires”, and “validity” also appeared. This indicates that there were many studies on oral health status in different populations, and that risk factors and validation of tools remained hot topics. In the recent years, “psychology” has been a popular burst keyword, indicating that research on the development of an instrument for psychometric evaluation may be one of the frontiers in the field of OHL. The existing instruments to evaluate OHL use two main strategies: word recognition and reading comprehension.^44^ However, some aspects of oral and dental health literacy are ignored by the existing tools. Further work is therefore needed on a comprehensive OHL instrument for international use, ensuring that it is both simple and brief.^45^ A comprehensive, valid, and reliable scale could assist in identifying factors affecting oral health.

### Strengths and limitations

To our knowledge, this is the first study to systematically analyze the research on OHL using the scientometric method. This study provided an insight into OHL and valuable information for OHL researchers to identify new perspectives on potential collaborators, cooperative countries, hotspots, and future research directions. However, this study also has some limitations. The scientometric analysis was based on papers obtained from the WoSCC and Scopus databases to meet the reference format requirements of the CiteSpace software. Although these databases embrace a relatively comprehensive range of studies, it would be better if more databases had been included for the bibliographic analysis. Furthermore, most countries in the world have their own database resources using different languages. Therefore, it is hoped that future studies will analyze and compare a range of different databases in the OHL area.

## Conclusion

The field of OHL is currently thriving, and we expect this to continue. The development of an OHL instrument and health promotion practice are current focus areas, and the development and psychometric evaluation of a comprehensive OHL instrument may be the next frontiers in the field of OHL. Strategic cooperation among countries, core authors, institutions, hospitals, and communities should be encouraged so that resources can be shared to promote the development of OHL and address problems in the field.

## Data Availability

All data sets generated for this study are included in the paper.
